# 

**Published:** 1888-04

**Authors:** 


### BOOKS AND PAMPHLETS RECEIVED.

A MONOGRAPH ON SURRA, OR PERNICIOUS ANÆMIA IN THE LOWER ANIMALS. By R. W. Burke, A. D. V. Jubbulpore, 1888.

SHOULD PHYSICIANS BE PHARMACISTS. By Charles L. Mitchell, M. D. Reprint from the Philadelphia Medical Times.

THE GALVANO-CAUTERY SOUND AND ITS APPLICATION, ESPECIALLY IN HYPERTROPHY OF THE PROSTATE. By Robert Newman, M. D., Reprint from the New England Medical Monthly.

SYNOPSIS OF THE SECOND HUNDRED CASES OF URETHRAL STRICTURE, TREATED BY ELECTROLYSIS. By Robert Newman, M. D. Reprint from the Journal of the American Medical Association.

TRANSACTIONS OF THE AMERICAN DERMATOLOGICAL ASSOCIATION. 11th Annual Meeting.

THIRD ANNUAL REPORT OF THE NORTH TEXAS HOSPITAL FOR THE INSANE, at Terrill, Austin, 1887.

REMARKS ON CLASSIFICATION OF VERTEBRATA. By Prof. B. G. Wilder. Reprint from American Naturalist.

COMPARATIVE DATA FROM 2,000 INDIAN CRANIA IN THE U. S. ARMY MEDICAL MUSEUM. By R. W. Shufeldt, M. D. Reprint from Journal of Anatomy and Physiology. Vol. XXII.

THE LOMB PRIZE ESSAYS. Published by the American Health Association. Concord, 1886.

### AMERICA.

ALBANY.—Medical Annals.

AUGUSTA.—The Sanitary Inspector.

BALTIMORE.—Johns Hopkins University Circulars.

CAP ROUGE.—Le Naturaliste Canadien.

CHARLESTON.—Proceedings Elliot Society of Science and Art. August, 1886—July, 1887.

CHICAGO.—Weekly Drovers' Journal, National Live Stock Journal, The Chicago Tribune.

CINCINNATI.—Journal of the Cincinnati Society of Natural History. Vol. X., No. 4.

DAVENPORT.—Proceedings of the Davenport Academy of Natural Sciences. Vol. IV., 1882–84.

DETROIT.—The Microscope.

KNOXVILLE.—Agricultural Science.

NEW YORK.—American Veterinary Review, New York Medical Journal, Medical Analectic, Scientific American, Forest and Stream, Turf, Field and Farm.

PHILADELPHIA.—Medical and Surgical Reporter, Therapeutic Gazette, American Naturalist, Reins and Whip.

RED-WING.—Public Health in Minnesota. Vol. III., Nos. 8–11.

ST. LOUIS.—Weekly Medical Review.

WILMINGTON.—Delaware Farm and Home.

### ASIA.

BATAVIA.—Verslag van de Directie der Vereeniging tot Bevordering van Veeartsenijkunde in Nederlandsch-Indie over de jaren, 1884–87.

BOMBAY.—Journal of the Bombay Natural History Society. Vol. II. No. 4.

MADRAS.—The Quarterly Journal of Veterinary Science in India.

### AUSTRALIA.

MELBOURNE.—Report of the Zoological and Acclimatization Society of Victoria, 1886.

SYDNEY.—Proceedings of the Linnean Society of New South WALES. Vol. II., Pt. 3. List of the names of contributors to the first series vol. I.-IX. of Proceedings of Linneau Society of New South Wales.

### AUSTRIA.

VIENNA.—Verhandlungen der K. K. Zoologisch-botanischen Gesell schaft in Wein. Band XXXVII. jahrgang 1887. Monatschrift des Vereius der Thierarzte in Œsterreich. 1887.

### BELGIUM.

GHENT.—Annals et Bulletin de la Societie de Medicine de Gand.

### FRANCE.

PARIS.—Le Progress Medical.

TOULOUSE.—Revue Veterinarie.

### GERMANY.

AUGUSBURG.—Wochenschrift fur Theirheilkunde und Viehzucht.

BERLIN.—Archiv fur Wissenschaftliche und Praktische Thierheilkunde.

BREMEN.—Abhandlungen herausgegeben von Naturwissenschaftlichen Vereine zu Bremen. Band IX. Heft 4.

FRANKFORT.—Der Zoologische Garten.

MUNSTER.—Jahres-Bericht der Zoologischen Sektion des Westfalischen Provinzial-Vereins fur Wissenschaft und Kunst. 1886-7.

### GREAT BRITAIN.

LONDON.—The Veterinary Journal. The Veterinarian.

### ITALY.

MILAN.—La Clinica Veterinaria.

PADOVA.—Atti della Societa Veneto-Trentina di Scienze Naturali. Vol. X. Fas. 1.

PAVIA.—Bolletino Scientifico. Vol. IX. No. 3.

PISA.—Giornale de Anat. Fisiol, e Patol, Degli Animali.

### RUSSIA.

ST. PETERSBURG. The Archives of Veterinary Science.

SPAIN.

**Madrid.**—Gaceta Medico-Veterinaria.
**Valencia.**—La Cronica Medica.

SWEDEN.

**Stockholm.** Tidskrift fur Veterinar-Medicine.

SWITZERLAND.

**Zurich.**—Schweizer-Archiv fur Theirheilkunde.

